# Supplementary material for: The synchronicity of COVID-19 disparities: Statewide epidemiologic trends in SARS-CoV-2 morbidity, hospitalization, and mortality among racial minorities and in rural America
Source: PLoS One. 2021 Jul 23;16(7):e0255063. doi: 10.1371/journal.pone.0255063 (PMC8301667; doi:10.1371/journal.pone.0255063)
Supplement: Attachment — Submitted filename: Review_Plos_One_15March21.docx [file pone.0255063.s001.docx]

In the submitted manuscript, the authors studied the infection rates, mortality, and hospitalizations from SARS-Coronavirus-2 disease in the state of Indiana from March 1, 2020, through December 31, 2020. In their analysis, the authors classified the disease outcomes based on race, age, gender, and residence designated by rural/urban status. The authors compared three phases based on social distancing and other state policies. The modality of data analysis is descriptive. I agree with the authors that longitudinal/time series analysis of Covid-19 is limited in the literature, however, emerging. I felt the analysis, the results, and the conjectures did not elevate the manuscript to a level with substantial innovation.

There is plenty of published work on the racial disparities and the effect of social determinants of health on Covid-19. Many of those works used rigorous data analytic approaches. I am including a few of them here:

1. Millett GA, Jones AT, Benkeser D, et al. Assessing differential impacts of COVID-19 on black communities. Ann Epidemiol. 2020;47:37-44.
2. DiMaggio C, Klein M, Berry C, Frangos S. Blacks/African Americans are 5 times more likely to develop COVID-19: spatial modeling of New York city ZIP code-level testing results. Ann Epidemiol. 2020;51:7-13

There is substantial work done assessing rural/urban differences as well, for example

1. Paul, R., Arif, A., Pokhrel, K., & Ghosh, S. (2021). The association of social determinants of health with COVID‐19 mortality in rural and urban counties. The Journal of Rural Health. <https://doi.org/10.1111/jrh.12557>
2. Karim, S. A., & Chen, H. F. (2021). Deaths From COVID‐19 in Rural, Micropolitan, and Metropolitan Areas: A County‐Level Comparison. The Journal of Rural Health, 37(1), 124-132.
3. Peters, D. J. (2020). Community susceptibility and resiliency to COVID‐19 across the rural‐urban continuum in the United States. The Journal of Rural Health, 36(3), 446-456.

Similar analyses presented in the submitted manuscript can be easily available on many state and county websites, for example, see Indiana (<https://www.coronavirus.in.gov/2393.htm>) and North Carolina (<https://covid19.ncdhhs.gov/dashboard/cases-demographics>).
