# Supplementary material for: The synchronicity of COVID-19 disparities: Statewide epidemiologic trends in SARS-CoV-2 morbidity, hospitalization, and mortality among racial minorities and in rural America
Source: PLoS One. 2021 Jul 23;16(7):e0255063. doi: 10.1371/journal.pone.0255063 (PMC8301667; doi:10.1371/journal.pone.0255063)
Supplement: Attachment — Submitted filename: PONE Response to Reviewers - FINAL - 20210507.DOCX [file pone.0255063.s002.DOCX]

**Dear Editors,**

Thank you for the opportunity to further revise our manuscript (PONE-D-21-05556) entitled *"The COVID-19 health equity twindemic: Statewide epidemiologic trends of SARS-CoV-2 outcomes among racial minorities and in rural America."*

Below, we document how we have addressed each issue raised by the reviewers on a point-by-point basis. Overall, we greatly appreciate the enthusiasm for our work and for the enriching comments we have received.

We believe the manuscript is strengthened by the suggestions and feedback. We genuinely appreciate the opportunity to have our work considered by *PLOS One*.

Sincerely,

Brian Dixon, PhD
Corresponding Author

| Reviewer #1 | Authors’ Response |
| --- | --- |
| The modality of data analysis is descriptive. I agree with the authors that longitudinal/time series analysis of Covid-19 is limited in the literature, however, emerging. I felt the analysis, the results, and the conjectures did not elevate the manuscript to a level with substantial innovation.  There is plenty of published work on the racial disparities and the effect of social determinants of health on Covid-19. Many of those works used rigorous data analytic approaches. I am including a few of them here:  1. Millett GA, Jones AT, Benkeser D, et al. Assessing differential impacts of COVID-19 on black communities. Ann Epidemiol. 2020;47:37-44.  2. DiMaggio C, Klein M, Berry C, Frangos S. Blacks/African Americans are 5 times more likely to develop COVID-19: spatial modeling of New York city ZIP code-level testing results. Ann Epidemiol. 2020;51:7-13 | Given the suggestions from Reviewer 2, we have added more robust statistical models in the revised paper to address concerns around the paper being too descriptive.  With respect to the cited papers, the prior analyses did not use individual-level data for their modeling of disease trends. Instead they relied on census-level data on the proportion of black/African American residents for a given area compared to overall COVID-19 cases. These population-level indicators are useful, but they have a number of limitations. For example, even though an area is majority Black does not mean all COVID-19 cases in that area will be among Black individuals. Furthermore, they did not measure the % of COVID-19 cases among blacks/AA. They simply used total counts associated with county statistics. Our data are at the individual level, and we count the actual number of black/African American residents in an area and divide by the census # of black/AA residents. Furthermore, both of the cited articles use data thru April 2020. Prior analyses therefore do not account for the continued evolution of the pandemic throughout 2020. Our data and timeframe are both novel, adding to the knowledge base around the impact of COVID-19 on the Black community.  We added critiques of the prior literature to the Introduction section of the paper. |
| There is substantial work done assessing rural/urban differences as well, for example  1. Paul, R., Arif, A., Pokhrel, K., & Ghosh, S. (2021). The association of social determinants of health with COVID $B!> (J19 mortality in rural and urban counties. The Journal of Rural Health. https://doi.org/10.1111/jrh.12557 (J (J2. Karim, S. A., & (J (JChen, H. F. (2021). Deaths From COVID $B!> (J19 in Rural, Micropolitan, and Metropolitan Areas: A County $B!> (JLevel Comparison. The Journal of Rural Health, 37(1), 124-132. (J (J3. Peters, D. J. (2020). Community susceptibility and resiliency to COVID $B!> (J19 across the rural $B!> (Jurban continuum in the United States. The Journal of Rural Health, 36(3), 446-456. | The studies cited on rural areas have similar limitations as the ones cited on Black populations. The paper from Peters (2020) used data from the American Community Survey (ACS) to examine whether a county was susceptible to COVID-19. This paper did not measure actual rates of infection or mortality. The paper from Karim and Chen (2020) used county-level census data associated with overall trends in cases and deaths. The paper only included data thru June 2020, well ahead of the fall surge that impacted rural areas. The paper from Paul et al. (2021) was published a few days before we submitted our article. Furthermore, this paper examines mortality data through October 2020. Given that deaths lag cases and hospitalizations, many of the deaths in rural areas were not reported until the end of 2020, even into March 2021 (e.g., Ohio, Kentucky, and Illinois reported sizable data backlogs in deaths in early 2021). Our analysis used more complete data for a longer period of time with data from individuals rather than just county indicators. The dataset as well as the timeframe increase the novelty of our paper. Furthermore, our findings also differ from those of Karim and Chen which found rates to be lower in rural areas. The increases this past fall demonstrate rural areas did not heed the warnings with significant impact on rural populations in terms of hospitalization and death.  We added these citations and critiques of them to the Introduction section of the paper. None of these citations suggest our analysis is useless. |
| Similar analyses presented in the submitted manuscript can be easily available on many state and county websites, for example, see Indiana (https://www.coronavirus.in.gov/2393.htm) and North Carolina (https://covid19.ncdhhs.gov/dashboard/cases-demographics). | We acknowledge that many state health departments are reporting information about COVID burden by race on their public dashboards. However, in this paper we adjust for the population of Blacks, which is typically not performed on the websites. For example, the Indiana site reports the % of individuals testing positive for COVID-19 based on the # tests performed (denominator), not the proportion of Black individuals living in Indiana. The % population figures presented in addition come from the Census and have nothing to do with COVID-19. They are there for comparison but these % cannot be directly compared. These data actually skew reality for the casual observer of the website, minimizing the true impact on the Black community. |
| Reviewer #2 | Authors’ Response |
| The paper needs thorough editing to avoid clunky sentences.  See example in line 57: There further exists little evidence on individuals in rural communities | We have fixed the sentence on line 57 and edited the paper for clunky sentences. |
| There are also typos and other grammatical issues. Please proofread carefully. | We have given the revised paper careful review for grammatical issues. |
| What are the demographic characteristics of Indiana? This information can help you set the relevance of this paper. Particularly in reference to the % of the population living in rural areas and minority groups. | We added demographic information on residents to the Setting sub-section in the Methods section. The prior version of the paper contained information on the % of the population living in rural areas. |
| Methodologically, the analysis is largely descriptive. I advise the authors to run some regressions (or even t-tests and ANOVAs) to tell us if the differences between minorities v. whites and rural v. urban are statistically significant. | As suggested, we conducted ANOVAs to examine the significance of differences by demographic groups across phases. In the revised manuscript, we added p-values to Table 2, and we comment on the significance of the differences by age, race, sex, and geography. |
| Add those who classify themselves as others racially to the analysis and graphs since you have a hunch they are largely Hispanics. Add to the figures. | As suggested, we added the Other race category to Figure 1. |
| I don’t agree with the twindemic characterization. The race and geography risk should be framed as compounded risk factors rather than a twindemic. A twindemic implies two simultaneous health issues, like COVID and mental disorders. | We agree that our use of the term ‘twindemic’ is not textbook. We believe that disparities stemming from systemic racism are a disease plaguing the American health care system. However, we did change the paper title and references from twindemic to synchronicity as this better reflects the simultaneous nature of these two distinct sets of disparate outcomes among Black/Brown and rural populations. |
| In the limitations, when you say: The patterns observed in Indiana may not generalize to all geographic regions of the U.S. or other countries (276-277) - stress that these patterns might be more applicable to states with similar proportions of the population living in rurality and with comparable rates of minority populations (see second comment). | Thank you for this suggestion. We have revised this section accordingly in the paper. |
| 282 – Correct mitigation (it says mitgation) and say mitigation strategies because the sentence is repetitive: (282-283) $B!H (Jthis suggests aggressive mitgation for a longer period of time may be necessary for stronger mitigation $B!I (J. (J (J286: I think there is a missing word here $B!H (JAs public health attempts to mitigate disease spread going forward $B!I (J | We have revised this section of the paper. Thank you for the suggested edits. |
| 302 – I think you mean you can achieve equity in vaccinating | Thanks for pointing out this error. We have corrected the sentence. |
| 306 – Higher morbidity and mortality (add higher) | This suggested change was made to the manuscript. |
